# Establishment of a novel experimental system for studying the photoperiodic response of short-day dicots using soybean ‘cotyledon-only plant’ as material

**DOI:** 10.3389/fpls.2022.1101715

**Published:** 2023-01-06

**Authors:** Chunlei Zhang, Xin Xu, Fulu Chen, Shan Yuan, Tingting Wu, Bingjun Jiang, Enoch Sapey, Cunxiang Wu, Shi Sun, Changhong Guo, Tianfu Han

**Affiliations:** ^1^ College of Life Science and Technology, Harbin Normal University, Harbin, China; ^2^ Ministry of Agriculture and Rural Affairs (MARA) Key Laboratory of Soybean Biology (Beijing), Institute of Crop Sciences, The Chinese Academy of Agricultural Sciences, Beijing, China; ^3^ Council for Scientific and Industrial Research (CSIR)-Oil Palm Research Institute, Kade, Ghana

**Keywords:** soybean, cotyledon-only plant, photoperiodic response, model material, dicot

## Abstract

Soybean is an important model crop for photoperiodic response studies in plants and contributes significantly to the study of plant development and physiology in the past century. Because soybean plant is much bigger in size and longer in life cycle than *Arabidopsis*, it needs much more space for growth and time for investigation, which significantly hamper the efficiency of research. In the current study, we tested the photoperiodic response of a distinctive artificially-made cotyledon-only plant (COP) using a photoperiod-sensitive soybean variety Zigongdongdou (ZGDD) and other varieties with diverse sensitivity to photoperiod. ZGDD COPs flowered 39.4 ± 2.5 d after emergence under short-day conditions but maintained vegetative growth under long-day and night break conditions, which is similar to the case in the intact ZGDD plants. The COPs of early-maturing and medium-maturing soybean varieties also grew and flowered normally under natural day-length conditions. At the molecular level, the key genes in the photoperiodic pathway such as *E1*, *GmFT1a*, *GmFT2a*, and *GmFT5a* in the COPs also showed the same photoperiod sensitivity as in the intact plants. In addition, a simpler material of COP with only one cotyledon and root was generated and found to be sensitive to photoperiod as well. Notably, the COPs are only one-fifth the height of intact plants and one-third the maximum diameter of the intact plants grown in chambers 30 d after emergence. Based on COPs, we established a novel experimental system characterized by an entire photoperiodic response and longer longevity of cotyledons in addition to small plant size, ensuring the consistency, reliability, and stability of plant materials. COPs have the potential to be a novel model material for studies of the developmental biology of soybean and other dicots.

## Introduction

Soybean (*Glycine max*) is a typical short-day plant that is sensitive to photoperiod. It was one of the two species used in the discovery of photoperiodism in plants (tobacco is another) and made a great contribution to the basic theoretical developments in the photoperiodic response (Garner and Allard, 1920; Parker et al., 1946). To date, soybean is still a model species for photoperiodic response studies (Zhang et al., 2020; Ort et al., 2022). Using soybean as material, it was found that the leaf is the main organ that perceives photoperiodic signals (Heinze et al., 1942), Short-days (SDs) promote the flowering and maturity of soybean and long-days (LDs) conditions suppress these processes (Garner and Allard, 1920; Han et al., 2006), the dark period is the core stage evidenced by night break treatment with white or red/far-red light (Downs, 1956; Coulter and Hamner, 1964), etc. Recently, unique effect of LD on soybean flowering and post-flowering was identified (Jiang et al., 2011). When soybean is planted in SD for floral induction and then transferred to LD, it will reverse to vegetative growth along with flower and pod falling. This is termed ‘flowering reversion’ (Han et al., 1998; Wu et al., 2006).

In addition to the progresses in physiology, advances in molecular biology underlying the photoperiodic pathway have been achieved in soybean (as reviewed by Lin et al., 2021). In this pathway, the photoreceptors E3 (GmPHYA3) (Watanabe et al., 2009) and E4 (GmPHYA2) (Liu et al., 2008) receive light signals and transmit them to circadian clock factors, such as E2 (GmGI; Watanabe et al., 2011; Dong et al., 2022; Wang et al., 2022), GmLUX (Bu et al., 2021; Fang et al., 2021), J (GmELF3; Lu et al., 2017; Yue et al., 2017), and GmPRR3a/3b or Tof11/12 (Li et al., 2020; Lu et al., 2020; Wang et al., 2020). Then, different oscillation rhythms of the circadian clock are formed in SD and LD and control the flowering time of soybean plants by regulating the E1-FT-AP1 module (Lin et al., 2021). The component of the evening complex J/LUX in the circadian clock together with the legume-specific factor E1 has been demonstrated to be a core in the photoperiodic response of soybean (Bu et al., 2021). Recently, the relationship between E1 and E3/E4 was further elucidated (Lin et al., 2022). In addition, several new genes including *QNE1* (Xia et al., 2022), *SOC1a* (Kou et al., 2022), *GmSPA3c* (Li et al., 2022b), *GmHY2* (Zhang et al, 2022) and *GmCDPK38* (Li et al., 2022a) were found to play important roles in the photoperiodic flowering pathway of soybean (Gupta et al., 2022), which may be potential targets for molecular breeding of soybean in the future.

Current developments in the physiology and molecular mechanism of the soybean photoperiodic response are distinguished mainly based on intact plants. However, soybean plants have a long-term process of growth, many leaves, and large plant sizes. These factors cause difficulty in the photoperiodic treatment of large numbers of plants and precise control of the environments. Additionally, due to the short longevity of different leaves, it is difficult to carry out long-term tracing experiments in a specific position (organ). These problems limit the efficiency and accuracy of photoperiodic studies. Due to the small plant size and short life cycle, *Arabidopsis thaliana* is a model plant that is widely used in the study of many biological processes of plants, suggesting that the reduced plant size is an ideal solution for exploring other plants for photoperiodic response studies.

Like leaves, cotyledons can perceive photoperiodic signals (Paton, 1971; Ogawa and King, 1990; Li and Tan, 1991; Yoo et al., 2013; Xu et al., 2021) and produce the floral-promoting gene *FLOWERING LOCUS T* (*FT*) to accelerate flowering in *Arabidopsis* (Yoo et al., 2013). This was also observed in soybean cotyledons (Xu et al., 2021). Importantly, soybean cotyledons have the ability to support the entire life cycle from emergence to maturation in a special cotyledon-only plant (COP) with only cotyledons as the main aboveground organ (Xu et al., 2021), showing that the COP could act as a model plant for studying the photoperiodic response.

In this study, we used COPs as the main material and identified the morphological characteristics, photoperiodic response, night-break treatment, and gene expression analysis of flowering-time related genes. Then, we generated a simpler COP with only one cotyledon and root, which was shown to be sensitive to photoperiods. Based on the COPs, we established a model experimental system for studying the photoperiodic response. This system will play an important role in developmental biology studies of soybean and other dicot species.

## Materials and methods

### Plant materials

The soybean varieties Heihe27 (HH27, maturity group (MG) 0), Heihe43 (HH43, MG 0), Zigongdongdou (ZGDD, MG VIII), Zhonghuang30 (ZH30, MG III), and Zhonghuang39 (ZH39, MG III) were used in this study. HH27 and HH43 are photoperiod-insensitive and early-maturing soybean varieties. ZH30 and ZH39 are photoperiod-sensitive and medium-maturing soybean varieties. ZGDD is a photoperiod-sensitive and late-maturing soybean variety (Han et al., 1998; Wu et al., 2006).

### Generation of cotyledon-only plants

For the generation of COP, soybean seeds were planted in growth chambers at 26°C. All the organs above the cotyledonary node were removed at VC (unifoliolate leaf stage). Subsequently, the newly-produced leaves or stems at the cotyledonary node were removed soon when they began to emerge (**Supplementary Figure S1**).

For the generation of COP with only one cotyledon and root, soybean seeds were sterilized with chlorine gas for 16 h and then grown on germination culture medium (Chen et al., 2018). For hydroponic culture, the germinated seeds were cut into two half-seeds (**Supplementary Figure S2**), and the buds at the cotyledonary node on the half-seeds were removed and then grown in the Hoagland solution.

### Growing conditions and sample preparation

To analyze the flowering time of COP of different soybean varieties, ZGDD and HH27 COPs were grown in chambers at 26°C under short-day (SD, 12 h light/12 h dark) and long-day (LD, 16 h light/8 h dark) conditions. ZH39, ZH30, HH27, and HH43 COPs were grown in the natural day-length environment in Beijing, China. The sowing dates of HH27, HH43, and ZH39 were 20 May, 21 May, and 30 June 2019, and ZH30 was 20 May, 21 May, and 15 July 2019.

To examine the effect of night break on COP, ZGDD COPs were grown in a chamber at 26°C under SD (12 h light/12 h dark) conditions. A 1-h period of white-light exposure was conducted in the middle of the dark period (**Supplementary Figure S3**). Samples were harvested after two weeks after the night break treatment.

To test the expression level of flowering-time related genes in COP, ZGDD COPs were grown in chambers at 26°C under SD (12 h light/12 h dark) and LD (16 h light/8 h dark) conditions. The cotyledons were sampled every 3 d from germination.

To test the expression level of *GmFT2a* in COP with only one cotyledon and root, ZGDD were grown in chambers at 26°C under SD (12 h light/12 h dark) and LD (16 h light/8 h dark) conditions. The cotyledons of 7-d-old COP with only one cotyledon and root were sampled at 4 h after light on.

### Gene expression analysis

RNA extraction, reverse transcription, and quantitative real-time PCR were performed as described in our previous study (Wang et al., 2020). The primers for *GmActin*, *GmFT2a*, *GmFT5a*, and *E1* were described previously (**Supplementary Table S1**; Xu et al., 2021). The primers for *GmFT1a* are as follows: qGmFT1a-F: 5’- ATTCCTGCAACTACAGGGGC-3’, qGmFT1a-R: 5’-AAGTACATGGCCGCTACTGG -3’.

### Measurement of plant height and maximum diameter

The plant height is the distance from the cotyledonary node to the stem tip. The maximum diameter of the COP is the distance between the ends of the two cotyledons and the maximum diameter of the intact plant is the maximum distance between the two largest leaves in the horizontal plane.

### Phenotyping and statistical analysis

The flowering time was calculated from emergence (VE) to R1 (i.e., beginning bloom: one open flowering at any node on the main stream) (Fehr and Caviness, 1977). The statistical analysis was carried out in Microsoft Excel, and Student’s *t*-tests were used to assess the significance of differences.

## Results

### The plant size and cotyledon longevity of the cotyledon-only plants

**Figure 1:**
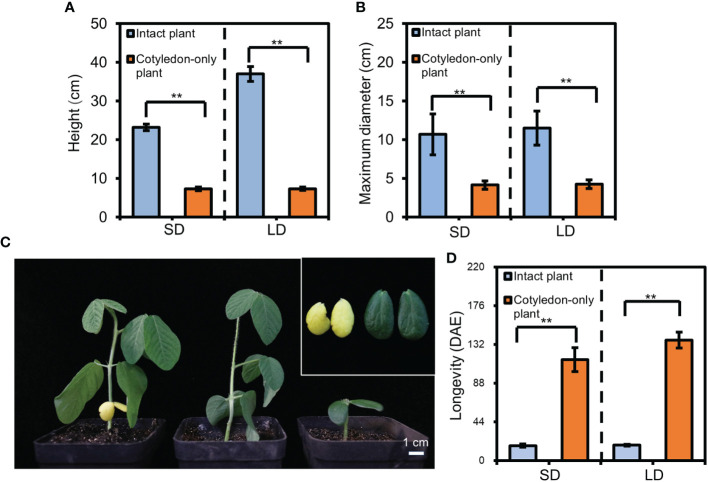
The height, maximum diameter, and cotyledon longevity of cotyledon-only plants (COPs) and intact plants. **(A)** The plant height for ZGDD COPs and intact plants. **(B)** The maximum diameter for ZGDD COPs and intact plants. Five plants were calculated in each experiment in **(A)** and **(B)**. **(C)** Comparison of cotyledons from the intact plant and COPs of ZGDD. The left plant is the intact plant with yellow cotyledons, the middle is the intact plant without cotyledons, and the right is the COP. A close-up view of the cotyledons from the intact plant and COP in C is shown in the top right. The photos in **(C)** were taken from 16-d-old plants. **(D)** The longevity of cotyledons from the COPs and intact plants of ZGDD grown under SD and LD conditions. Fifteen plants were included in **(D)**. DAE indicates d after emergence. Error bars indicate the standard deviation. Double asterisks indicate an extremely significant difference (*t*-test, *p* value < 0.01).

The COPs only contain two cotyledons as the main aboveground organs (Xu et al., 2021). To compare the plant size between the COPs and intact plants, we grew the COPs and intact plants of photoperiod-sensitive and early-maturing soybean variety ZGDD in chambers under SD and LD conditions and investigated the plant height and maximum diameter of 30-d-old plants. Under SD conditions, the plant height for COPs was only 7.3 ± 0.4 cm, which was significantly lower than that of the intact plants (23.2 ± 0.8 cm; **Figure 1A**). The maximum diameter for COPs (4.1 ± 0.5 cm) was also smaller than that for the intact plants (10.7 ± 2.6 cm; **Figure 1B**). Under LD conditions, we observed similar patterns in which the plant height and maximum diameter for COPs (7.5 ± 0.5 cm and 4.2 ± 0.6 cm, respectively) were significantly smaller than those for the intact plants (37.0 ± 1.9 cm and 11.5 ± 2.2 cm; **Figures 1A, B**). Thus, the COPs occupy less growing area than the intact plants.

Besides cotyledons, the intact plants contain multiple leaves compared to COPs. To identify the longevity of cotyledons in COPs and intact plants, we grew ZGDD in chambers under SD and LD conditions. The results showed that the longevity of cotyledons in intact plants was 16.9 ± 1.7 d and 17.4 ± 0.9 d under SD and LD conditions, respectively. However, the longevity of cotyledons in COPs was significantly increased to 114.7 ± 13.7 d and 136.9 ± 8.9 d under SD and LD conditions, respectively (**Figures 1C, D**), indicating that the COPs have longer cotyledon longevity than the intact plants.

### Photoperiodic responses of cotyledon-only plants

**Figure 2:**
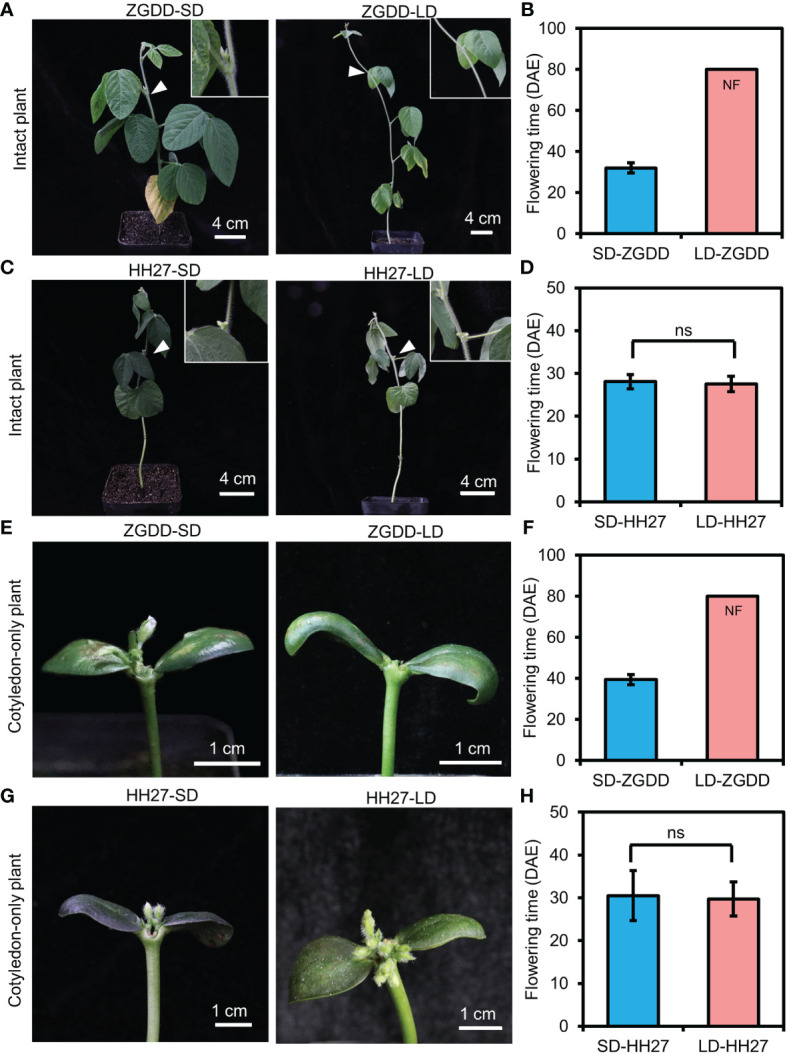
The flowering time of cotyledon-only and intact plants. **(A, C)** The flowering phenotype of Zigongdongdou (ZGDD; **A**) and Heihe27 (HH27; **C**) intact plants under short-day (SD, 12 h light/12 h dark) and long-day (LD, 16 h light/8 h dark) conditions. A close-up view (white arrows) of the floral bud and none floral bud is shown in the top right. **(B, D)** The flowering time of ZGDD **(B)** and HH27 **(D)** intact plants under SD and LD conditions. **(E**, **G)** The flowering phenotype of ZGDD **(E)** and HH27 **(G)** cotyledon-only plants (COPs) under SD and LD conditions. **(F, H)** The flowering time of ZGDD **(F)** and HH27 **(H)** COPs under SD and LD conditions. NF means no flowering during the experiment. Fifteen plants were calculated **(B)**, **(D)**, **(F)**, and **(H)**. DAE indicates the days after emergence. Error bars indicate the standard deviation. ns means not significant.

Previous studies have shown that COPs are responsive to photoperiods (Xu et al., 2021). To confirm the photoperiodic response of COPs, we compared the flowering time of COPs and intact plants of ZGDD, and the photoperiod-insensitive and early-maturing variety HH27 grown in chambers under both SD and LD conditions. For the photoperiod-sensitive variety ZGDD, intact plants flowered at 32.0 ± 2.5 d after emergence (DAE) under SD conditions and maintained vegetative growth under LD conditions (**Figures 2A, B**). Similarly, the COPs only flowered 39.4 ± 2.5 DAE under SD conditions and maintained vegetative growth under LD conditions (**Figures 2E, F**). For the photoperiod-insensitive variety HH27, the COPs and intact plants flowered normally both under SD (30.5 ± 5.9 and 28.1 ± 1.7 DAE) and LD (29.8 ± 4.0 and 27.6 ± 1.8 DAE) conditions (**Figures 2C, D, G, H**). The above results indicated that the COPs have the same photoperiodic response as intact plants.

To examine the photoperiodic response of COPs in the natural environment, we grew the COPs and intact plants of HH27 and another early-maturing variety HH43 belonging to MG 0, and medium-maturing varieties ZH30 and ZH39 belonging to MG III under natural day-length conditions in Beijing. Our results indicated that the COPs and intact plants of the above four varieties flowered normally (**Table 1**). Specifically, the flowering time of COPs flowered early when grown in the short day-length season and flowered late when grown in the long day-length season, which resembles that of intact plants (**Table 1**). These results showed that the COPs also respond to the natural photoperiod environment.

**Table 1:** The comparison of flowering time of intact and cotyledon-only plant of early-maturing and middle-maturing varieties under natural day-length conditions.

Variety	Date of sowing	Flowering time (DAE)		*p* Value
	(2020)	Intact plant	Cotyledon-only plant	
Heihe27	May 28	19.8 ± 1.2	29.0 ± 3.7	6.77E-07
	June 21	22.8 ± 0.6	30.5 ± 1.4	8.06E-11
	June 30	22.6 ± 0.6	28.6 ± 1.8	9.81E-09
Heihe43	May 28	19.5 ± 1.1	27.3 ± 4.7	7.11E-05
	June 21	22.6 ± 0.8	30.9 ± 2.7	3.19E-08
	June 30	22.1 ± 0.6	26.7 ± 1.7	2.04E-07
Zhonghuang30	May 28	27.9 ± 1.7	43.8 ± 4.1	1.60E-09
	June 30	30.3 ± 0.7	44.8 ± 2.5	7.10E-13
	July 15	27.4 ± 0.5	35.3 ± 1.5	5.39E-12
Zhonghuang39	May 28	42.4 ± 0.5	47.6 ± 3.2	7.15E-05
	June 21	39.2 ± 0.4	48.7 ± 1.9	7.24E-12
	June 30	36.3 ± 0.7	43.1 ± 1.3	1.61E-11

Values represent means ± standard deviation. Five plants were recorded for each treatment.

**Figure 3:**
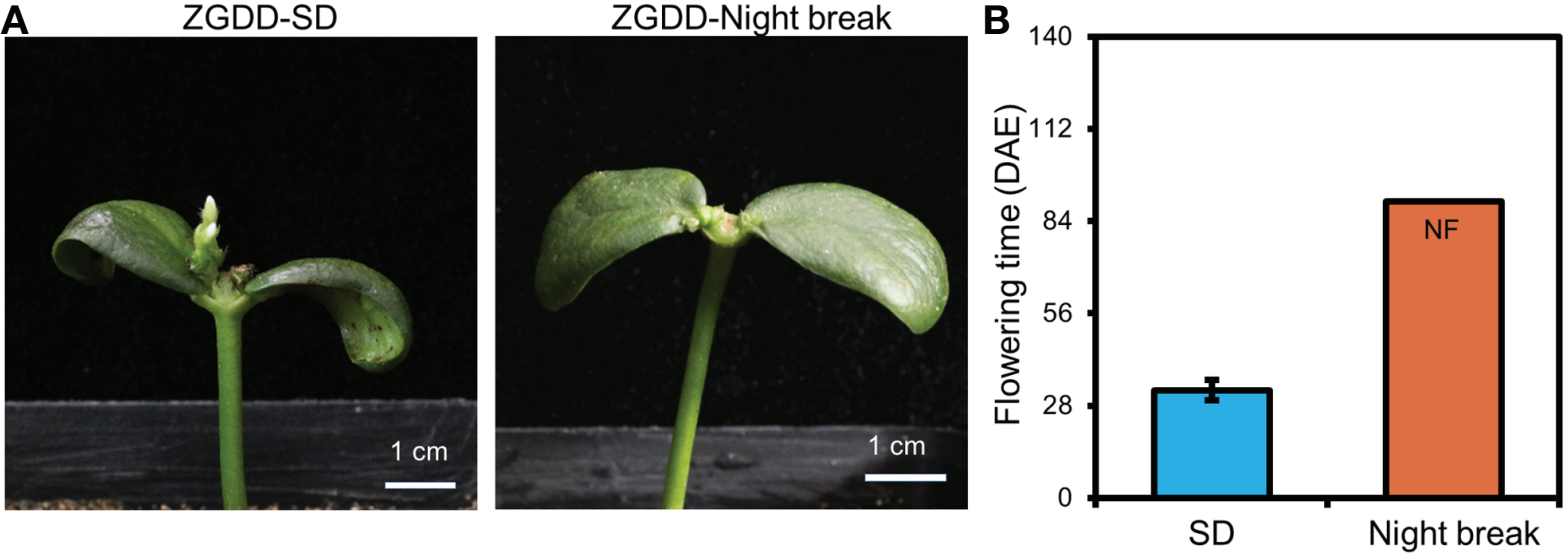
The flowering time of cotyledon-only and intact plants under night break treatments. **(A)** The phenotype of Zigongdongdou (ZGDD) cotyledon-only plants (COPs) treated with short-day (SD, 12 h light/12 h dark) and night break. **(B)** The flowering time of ZGDD COPs treated with SD and night break. DAE indicates d after emergence. NF means no flowering during the experiment. Fifteen plants were calculated in **(B)**. Error bars indicate the standard deviation.

The night break treatment acts as an important evidence of photoperiodic response and causes delayed flowering time of short-day plants (Xu et al., 2015). To test the effect of night break on the flowering time of COPs, the COPs and intact plants of ZGDD were planted under SD conditions with a night break treatment at midnight with 1-h exposure of white light. We found that both COPs and intact plants of ZGDD failed to flower after the night break treatment and maintained vegetative growth during the experiment (**Figures 3A, B**). Taken together, the COPs have a normal photoperiodic response similar to that of intact plants.

### The expression pattern of flowering-time genes in cotyledons of cotyledon-only plants

**Figure 4:**
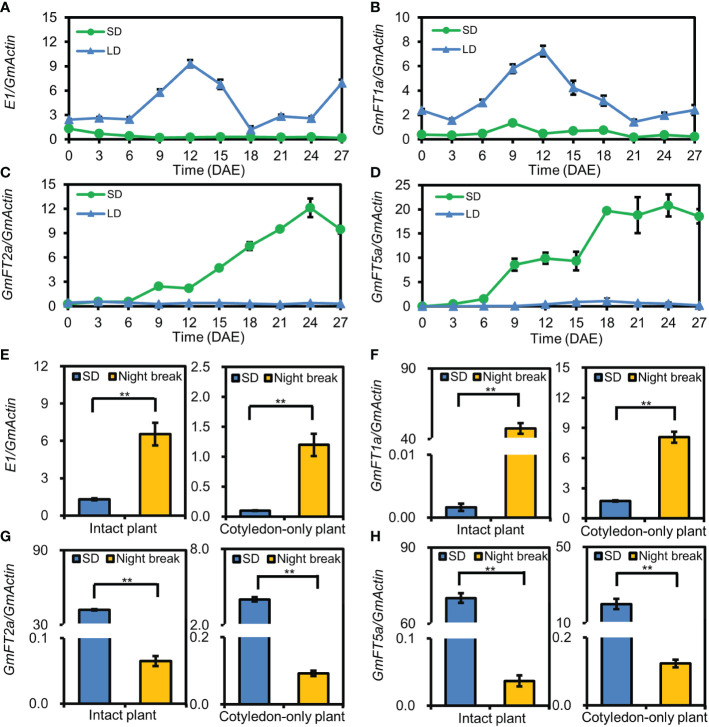
The expression pattern of flowering-time genes in cotyledon-only plants. **(A–D)** The expression pattern of *E1* **(A)**, *GmFT1a* **(B)**, *GmFT2a* **(C)**, and *GmFT5a* **(D)** in the cotyledons of Zigongdongdou cotyledon-only plants (COPs) under short-day (SD, 12 h light/12 h dark) and long-day (LD, 16 h light/8 h dark) conditions. The samples were harvested at 4 h after light on every three days from the emergence. Values represent the mean ± standard deviation from three biologically independent cotyledon samples from different plants. **(E–H)** The expression pattern of *E1* **(E)**, *GmFT1a* **(F)**, *GmFT2a* **(G)**, and *GmFT5a* **(H)** in the unifoliolate leaves of intact plants and cotyledons of COPs in SD and night-break. The samples were harvested at 4 h after light on. Values represent the mean ± standard deviation from three biologically independent unifoliolate or cotyledon samples from different plants. The reference gene is *GmActin*. Double asterisks indicate an extremely significant or significant difference (*t*-test, *p* value < 0.01).

*E1*, *GmFT1a*, *GmFT2a*, *GmFT5a* are among the key genes in the photoperiodic flowering pathway of soybean (Kong et al., 2010; Sun et al., 2011; Xia et al., 2012; Liu et al., 2018; Cai et al., 2020; Lin et al., 2021). To investigate the molecular mechanism involved in flowering time regulation in COPs, we examined the expression patterns of the above genes in the cotyledons of ZGDD COPs under SD and LD conditions. The results revealed that the floral-inhibiting genes *E1* and *GmFT1a* were significantly upregulated under LD conditions and downregulated under SD conditions, while the floral-promoting genes *GmFT2a* and *GmFT5a* were significantly upregulated under SD conditions and downregulated under LD conditions (**Figures 4A–D**). Furthermore, the expression levels of *E1*, *GmFT1a*, *GmFT2a*, and *GmFT5a* were also analyzed in ZGDD COPs and intact plants treated with a night-break. We found that the expression levels of *E1* and *GmFT1a* were significantly enhanced in the COPs and intact plants under the night-break treatment, while *GmFT2a* and *GmFT5a* were significantly repressed (**Figures 4E–H**). Taken together, the COPs present the same expression pattern of key genes in the photoperiodic flowering pathway as intact plants.

### Generation and characterization of cotyledon-only plants with only one cotyledon and root

**Figure 5:**
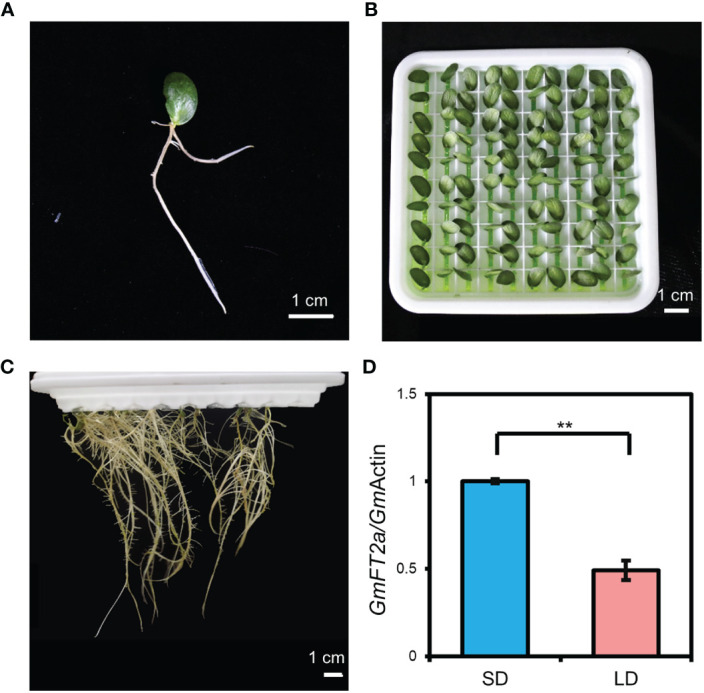
Generation of cotyledon-only plants (COPs) with only one cotyledon and root and their photoperiodic response. **(A)** One-week-old *in vitro* cotyledon with emerging root. **(B**, **C)** COPs with only one cotyledon and root under hydroponic culture: 4-d-old in **(B)** and two months old in **(C)**. **(D)** The expression level of *GmFT2a* in the cotyledons from one-week-old COPs with only one cotyledon and root through hydroponic culture under short-day (SD, 12 h light/12 h dark) and long-day (LD, 16 h light/8 h dark) conditions. The samples were harvested at 4 h after light on. Values represent the mean ± standard deviation from three biologically independent cotyledon samples from different plants. The reference gene is *GmActin*. Double asterisks indicate an extremely significant difference (*t*-test, *p* value < 0.01).

Using individual cotyledon, we generated a very simple cotyledon-only plant with only one cotyledon and root (**Figure 5A**). Specifically, a single cotyledon with a 0.2-cm petiole was grown in hydroponic medium. After approximately 7 d, the roots were grown from the petiole. The COP with only one cotyledon and root occupies a very small space size in which 100 plants can be grown in a 17 cm × 17 cm × 5 cm hydroponic tray (**Figures 5B, C**). We analyzed the expression level of *GmFT2a* in the cotyledon of COP with only one cotyledon and root to identify its photoperiodic response. The results showed that the expression level of *GmFT2a* was significantly upregulated under SD conditions compared with LD conditions (**Figure 5D**), suggesting that COP with only one cotyledon and root is also sensitive to photoperiod.

## Discussion

### Establishment of a novel experimental system based on cotyledon-only plants

Soybean is an important model plant for photoperiodic response studies. Many important discoveries in the photoperiodic response of plants have been interpreted using soybean as the material (Garner and Allard, 1920; Wu et al., 2006). However, intact soybean plants are not suitable for large-scale, rapid, and efficient experimental platforms and basic research due to the large space occupied by the plants and the long growth cycles.

*Arabidopsis thaliana* is the most-widely used model plant for biological study in plant, especially dicot species, due to its small plant size and short life cycle (Meyerowitz, 1989). For crop plants, the monocot rice (*Oryza sativa*) is widely used as a model material. In recent years, a new rice variety named ‘Xiaowei’ with small plant size was selected and demonstrated to be grown and screened in large numbers in the laboratory and even in the space station (Hu et al., 2018). This plant expedites the development of the physiology and molecular biology of crops. Similarly, the soybean variety ‘MiniMax’ also has a short plant height which can be used as a model plant (Qu et al., 2010a). Even though Xiaowei and MiniMax are relative smaller than the normal plants, they still have many leaves and big plant size compared to *Arabidopsis*.

In the current study, we generated a unique tiny plant, COP, with only the cotyledons, root, and hypocotyl that connected the cotyledons and root. Due to the totipotency cotyledons, this COP could complete the entire life processes of growth, development, and reproduction (Xu et al., 2021). It is the smallest version of plant species in nature reported to date. Moreover, COPs retain the photoperiod characteristics of the original variety and are amenable to the study of photoperiodism similar to normal plants. Based on this tiny plant, we established a novel COP experimental system for photoperiodic response studies in short-day plants and other dicot species. In addition, COPs are potential model materials that are applicable to other plant biology studies in addition to photoperiodic responses.

### Characteristics of the COP experimental system for the studies of photoperiodic responses

To date, many experimental systems have been established in soybean for photoperiodic response studies, including artificial light control, night-break, grafting experiments, and flowering reversion (Garner and Allard, 1920; Heinze et al., 1942; Downs, 1956; Wu et al., 2006; Qu et al., 2010b). Here, we propose a new COP experimental system that is an important complement to the current systems. Previous experimental systems mainly focus on the physiological and molecular changes of intact plants (Qu et al., 2010b; Zhang et al., 2020; Lin et al., 2021), while the COP experimental system facilitates a use of more accurately and stably controlled environments to trace biological changes in floral induction. Therefore, this novel system has unique characteristics compared to other experimental systems.

The smaller plant size of COP is the superior characteristic. Large numbers of COPs could be grown in a limited space with precise control of environmental factors, which is difficult to perform for intact plants with large plant sizes (Fang and Kong, 2022). COP has a pair of cotyledons as the main aboveground vegetative organ in its entire life cycle, while the intact plant has a longer life cycle and many leaf organs (Friedli and Walter, 2015). In so doing, the COP enables photoperiodic response studies only in the cotyledons and conduction of long-term tracking experiments, eliminating the interference of signaling and nutritional substances that arise from other leaves in the intact plants. The cotyledons of COP rapidly respond to photoperiods and express the floral-promoting gene *GmFT2a/5a* in SD and the floral-inhibiting genes *E1*/*GmFT1a* in LD, which resemble the intact plants (Kong et al., 2010; Sun et al., 2011; Xia et al., 2012; Liu et al., 2018; Cai et al., 2020). Thus, we could monitor the photoperiodic response at the molecular level without waiting for morphological changes, such as flowering, podding, and maturation. The cotyledons of COP are already full-development organs after emergence from soil under dark conditions (Cao et al., 2013), and are rich in nutrients such as protein, oil, and carbohydrates (Han et al., 2013; Wei et al., 2020). Hence, we could explore the beginning photoperiodic response, including light signal transduction and perception, day length discrimination, and gene regulation networks.

### Potential application of the COP experimental system in plant developmental biology

Apart from the photoperiodic response, the COP system could be applicable in other research fields. We noticed that the cotyledons could survive for more than 100 d in COPs, while they survived for only 15 d in the intact plants, suggesting that some signals arising from other leaves in the intact plants influence the senescence of cotyledons. It is worth considering the causes of this phenomenon, and it is possible to use this system to study the mechanism of leaf senescence. During the seedling stage, the cotyledons simultaneously undergo a complex process of nutrient degradation, transformation, and synthesis of photosynthetic products (Gonzalez and Vodkin, 2007; Han and Yang, 2015). That is, cotyledons act as both a source and a sink, which can be used to study physiological processes such as source-sink relationships and photosynthesis in plants.

In the tissue culture, regenerated buds are often faced with common problems in the rooting stage, such as no rooting, hardly rooting, and regenerated roots being polluted with bacteria. Due to the stronger root system and rich nutrition in cotyledons, we successfully used the COPs as stocks that are capable of supporting the survival of regenerated buds with problems in rooting as scions in the grafting experiment. Therefore, COPs will significantly increase the survival rate of regenerated buds in tissue culture and in turn elevate the efficiency of genetic transformation. Moreover, the simple COP with one cotyledon and root is cultured in the hydroponic medium. We could easily add hormones, mineral elements, and pesticides to the liquid and investigate the response of COP. In summary, the COP is an ideal experimental system for plant biology studies.

## Data availability statement

The original contributions presented in the study are included in the article/**Supplementary Material**. Further inquiries can be directed to the corresponding authors.

## Author contributions

TH and CG planned and designed the study. CZ and XX performed the experiments. SY, BJ and TW analyzed the data. CW and SS provided the technical support. CZ, XX, FC, ES, and TH wrote the manuscript. All authors contributed to the article and approved the submitted version.
