# Supplementary material for: Establishment of a novel experimental system for studying the photoperiodic response of short-day dicots using soybean ‘cotyledon-only plant’ as material
Source: Front Plant Sci. 2023 Jan 6;13:1101715. doi: 10.3389/fpls.2022.1101715 (PMC9853180; doi:10.3389/fpls.2022.1101715)
Supplement: Supplementary file 1 [file DataSheet_1.docx]

**Supplemental information**


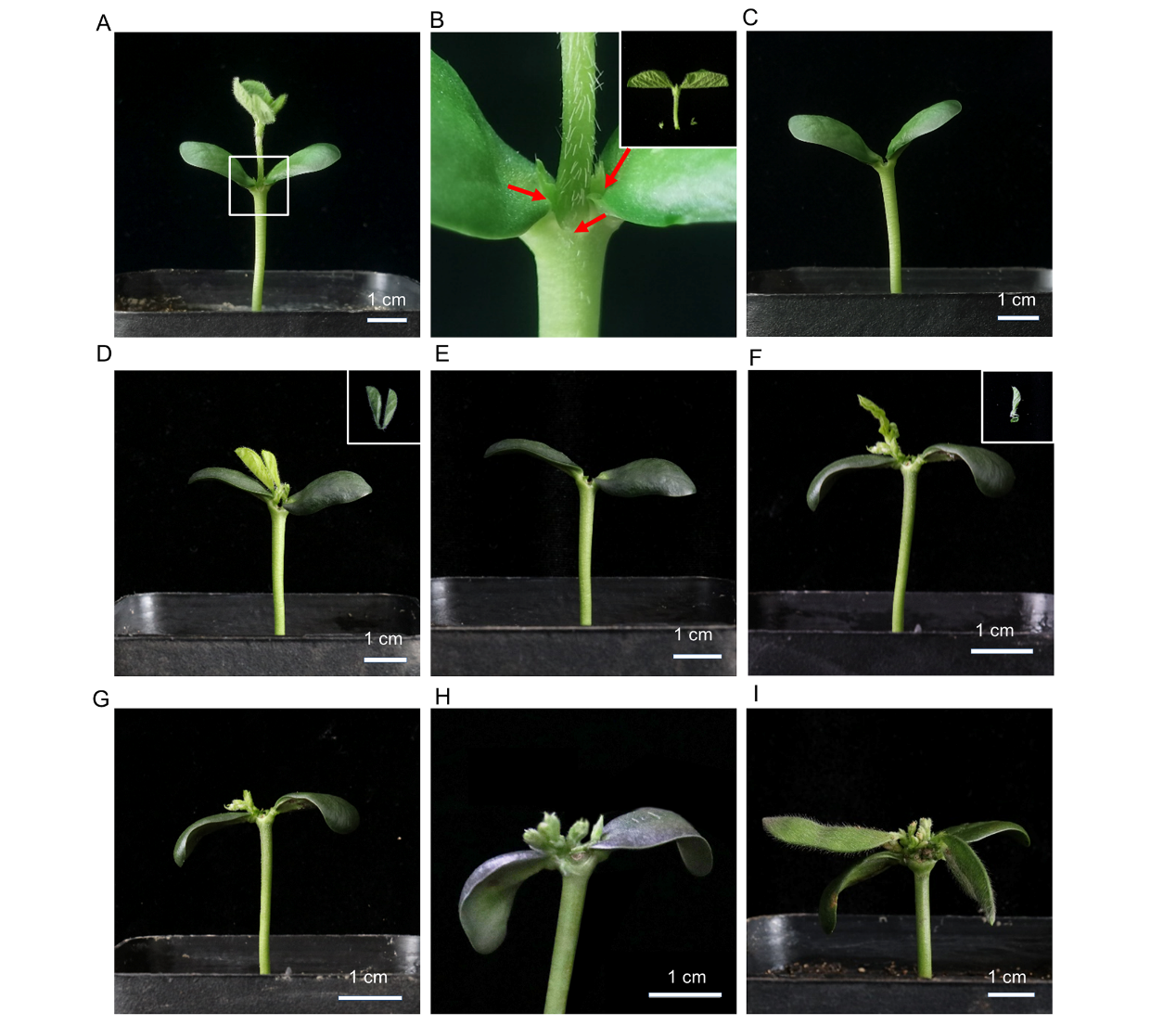


**Figure S1. The process for generation of cotyledon-only plant**

(A) A 3~5-d-old Heihe27 (HH27) seedling. (B) A close-up view of the area in the white box in (A) is shown. The arrows indicate the junction between epicotyl and cotyledon. The removed epicotyl and the shoots are shown in top right. (C) A 3~5-d-old HH27 COP after removal of the epicotyl and shoots. (D) 8~12-d-old HH27 COP. The removed newly emerged leaves are shown in top right. (E) 8~12-d-old HH27 COP after removal of the newly emerged leaves. (F) A 25~30-d-old HH27 COP with the floral bud and newly emerged leaves. The removed newly emerged leaves are shown in top right. (G) A 25~30-d-old HH27 COP with only floral buds. (H) A 28~38-d-old HH27 COP with flowers. (I) A 69-d-old HH27 COP with pods.


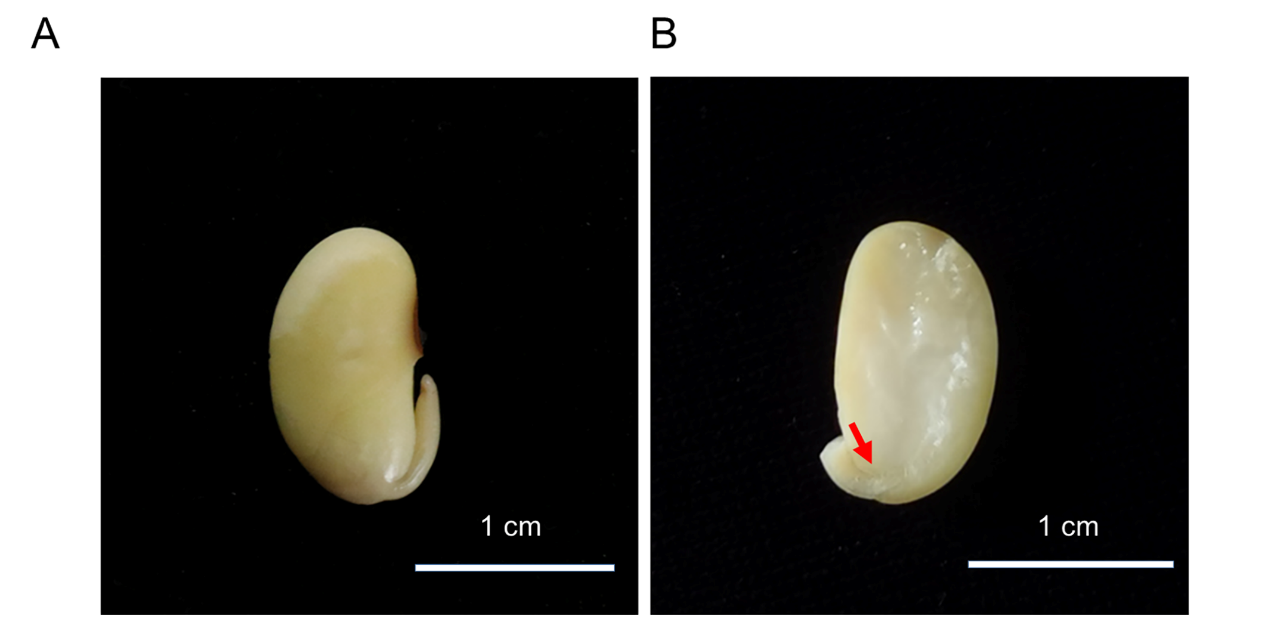


**Figure S2. The process for generation of cotyledon-root plant with only one cotyledon and root**

1. The seed germinated for 1 to 3 d in dark. (B) Separated the two cotyledons and removed the germ (red arrow) on the cotyledonary node.


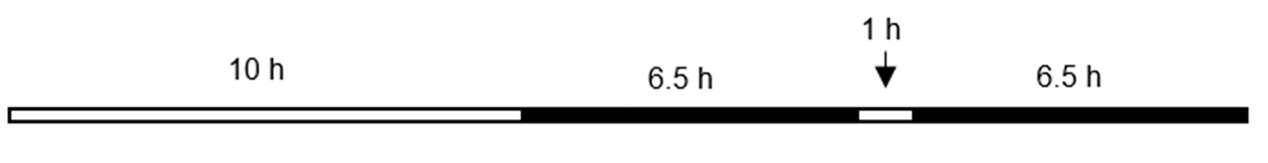


**Figure S3. Illustration of the night break treatment**

The white and black bars represent the light and dark cycles, respectively. The white bar below the arrow indicates the night break treatment. The numbers shown on the top of the bar represent the length of light and dark periods in hours.

**Table S1. The nucleotide sequences of the primers used in this study**

| Primer name | Primer sequence (5’ to 3’) |
| --- | --- |
| qGmActin-F | CGGTGGTTCTATCTTGGCATC |
| qGmActin-R | GTCTTTCGCTTCAATAACCCTA |
| qE1-F | CACTCAAATTAAGCCCTTTCA |
| qE1-R | TTCATCTCCTCTTCATTTTTGTTG |
| qGmFT2a-F | AAGTTGTCAACCAACCAAGGG |
| qGmFT2a-R | GAATCCCCATCATTGGTCTTGG |
| qGmFT5a-F | GATTGGGGATGTTCTCAACCCT |
| qGmFT5a-R | GTCTTCACCACCAACAGTAACCC |
